# Wnt5a Regulates the Assembly of Human Adipose Derived Stromal Vascular Fraction-Derived Microvasculatures

**DOI:** 10.1371/journal.pone.0151402

**Published:** 2016-03-10

**Authors:** Venkat M. Ramakrishnan, Kevin T. Tien, Thomas R. McKinley, Braden R. Bocard, Terry M. McCurry, Stuart K. Williams, James B. Hoying, Nolan L. Boyd

**Affiliations:** 1 Division of Cardiovascular Therapeutics, Cardiovascular Innovation Institute, University of Louisville, Louisville, Kentucky, United States of America; 2 Department of Physiology, University of Louisville, Louisville, Kentucky, United States of America; 3 Georgetown College, Georgetown, Kentucky, United States of America; 4 Division of Plastic Surgery, Department of Surgery, University of Louisville, Louisville, Kentucky, United States of America; University of Bari Medical School, ITALY

## Abstract

Human adipose-derived stromal vascular fraction (hSVF) cells are an easily accessible, heterogeneous cell system that can spontaneously self-assemble into functional microvasculatures in vivo. However, the mechanisms underlying vascular self-assembly and maturation are poorly understood, therefore we utilized an in vitro model to identify potential in vivo regulatory mechanisms. We utilized passage one (P1) hSVF because of the rapid UEA1+ endothelium (EC) loss at even P2 culture. We exposed hSVF cells to a battery of angiogenesis inhibitors and found that the pan-Wnt inhibitor IWP2 produced the most significant hSVF-EC networking decrease (~25%). To determine which Wnt isoform(s) and receptor(s) may be involved, hSVF was screened by PCR for isoforms associated with angiogenesis, with only *WNT5A* and its receptor, *FZD4*, being expressed for all time points observed. Immunocytochemistry confirmed Wnt5a protein expression by hSVF. To see if Wnt5a alone could restore IWP2-induced EC network inhibition, recombinant human Wnt5a (0–150 ng/ml) was added to IWP2-treated cultures. The addition of rhWnt5a significantly increased EC network area and significantly decreased the ratio of total EC network length to EC network area compared to untreated controls. To determine if Wnt5a mediates in vivo microvascular self-assembly, 3D hSVF constructs containing an IgG isotype control, anti-Wnt5a neutralizing antibody or rhWnt5a were implanted subcutaneously for 2w in immune compromised mice. Compared to IgG controls, anti-Wnt5a treatment significantly reduced vessel length density by ~41%, while rhWnt5a significantly increased vessel length density by ~62%. However, anti-Wnt5a or rhWnt5a did not significantly affect the density of segments and nodes, both of which measure vascular complexity. Taken together, this data demonstrates that endogenous Wnt5a produced by hSVF plays a regulatory role in microvascular self-assembly in vivo. These findings also suggest that manipulating Wnt signaling could enhance control of hSVF vascularization in tissue engineering applications.

## Introduction

Providing a mature microcirculation to engineered tissues remains a significant challenge in tissue engineering and regenerative medicine [1, 2]. To date, vasculatures have been engineered by using endothelial cells (EC) alone [3] or by including some kind of mesenchymal cell to stabilize the EC networks [4, 5]. Though these systems have provided tremendous insight [6–9], a more robust cellular vascularization system will be required to achieve successful long-term vascularization and engraftment of engineered tissue mimics. It is also critical to understand that a functional microcirculation will be required to meet the metabolic needs of the therapeutic parenchymal cells [10].

Adipose tissue is a unique endocrine and energy storage organ with the ability to increase or decrease in size, unlike most organs which are relatively weight stable [11]. Rupnick and colleagues proposed that adipose vasculature may be maintained in a relatively immature state, thereby allowing it to dynamically respond to tissue metabolic requirements in dynamic fashion [12]. While adipose tissue was originally digested to isolate adipocytes for study, it was also noted that the separated non-parenchymal cells, termed the stromal-vascular fraction cells (SVF), consisted of microvascular EC, perivascular cells, fibroblasts and leukocytes [13]. The component cells of SVF have been used as a source of EC [14] and multipotent mesenchymal cells (adipose stromal cells, ASC) [15]. ASC have been shown to produce multiple angiogenic growth factors and function as perivascular cells [16]. Therefore, non-parenchymal cells isolated from adipose tissue could provide a relatively accessible source of autologous therapeutic cells.

In spite of the significant work performed investigating the individual cells within SVF, the functional capabilities of the complete heterogeneous cell population [17–20] are less well-defined. We and others have demonstrated that implanted SVF spontaneously self-assembles into a hierarchical and perfused microvasculature in vivo [21–23]. This spontaneous self-assembly into microvasculatures requires the presence of EC, as their removal eliminates this activity [21, 22]. This suggests that this heterogeneous mix of cells interacts with each other to function as a cellular system. We utilized this vascular self-assembly capacity to support the vascularization of co-implanted model hepatocyte cells [22] and induced pluripotent stem cell-derived hepatocytes [24]. Though the endogenous cells within SVF possess an inherent capacity for providing a microcirculation, the mechanisms regulating this function are poorly defined.

Here we demonstrate that Wnt5a signaling regulates human adipose SVF microvascular self-assembly in vivo. Microvascular morphometric analysis indicates Wnt5a influences hSVF-EC proliferation while not significantly affecting vascular complexity (e.g. segment number and nodes). This suggests that Wnt5a could be useful for regulating the functional vascularization of hSVF for therapeutic applications.

## Materials and Methods

### Animals and Ethics Statement

All animal procedures were conducted in compliance with University of Louisville School of Medicine IACUC-approved protocols (IACUC #12060) and NIH guidelines. Animals were housed in a temperature- and light-controlled (13h light, 11h dark) ALAAC accredited facility. Food and water was provided ad libitum. Isoflurane gas was administered for anesthesia. Ketoprofen analgesic was delivered subcutaneously at the time of surgery and post-operatively every 24h for 2d. For the duration of the experiment, animals were observed daily for signs of discomfort, infection or distress. Regarding sacrifice, animals were anesthetized with Isoflurane gas until a surgical plane of anesthesia was reached before undergoing cervical dislocation and pneumothorax to allow for lacerating the heart and exsanguination.

### hSVF Isolation, Long-Term Storage, and General Culture

hSVF cells were isolated from the discarded lipoaspirate of three patients (identifying information was unavailable) obtained under IRB exemption #09.0037. The lipoaspirate was acquired and processed as previously described [22, 23]. The resulting cell pellet was divided and stored in liquid nitrogen. Thawed cells were resuspended in hSVF Growth Media (hSVF GM: M199, 1X L-glutamine and HEPES (Invitrogen), 10% FBS (VWR International), and EC growth supplement (developed in-house from bovine hypothalamus and augmented with heparin)) and plated into a 75cm^2^ NUNC tissue culture flask (Thermo-Fisher Scientific) pre-coated with 1% gelatin (Sigma-Aldrich). Cells were cultured overnight at 37°C and 5% CO_2_. A media change was performed the following morning to remove dead cells, with subsequent media changes performed every other day. Once confluent, hSVF cells were detached with 0.05% Trypsin-EDTA (Invitrogen).

### In Vitro Network Assembly

hSVF cells from passages 1 through 4 (P1 –P4) were plated at a density of 6x10^4^ cells/cm^2^ in hSVF GM. At days 1, 3, and 5 post-plating, all supernatant was aspirated and the cells were fixed in 1% paraformaldehyde (PFA; Electron Microscopy Sciences) for 10 minutes at RT before being washed with PBS (Invitrogen) for 5 minutes. All wells were labeled with Ulex Europaeus Agglutinin-1 (UEA1; Vector Laboratories) directly conjugated to fluorescein for human EC as well as DAPI nuclear dye (Thermo Fisher). Cells were imaged using an IX81 inverted fluorescence microscope (Olympus).

### Inhibitor Treatment

P1 hSVF cells were plated as described and allowed to adhere overnight. The following day, the media was supplemented with the Wnt inhibitor IWP2 (Tocris Biosciences) (at 0, 6.25, 12.5, and 25μM; also see S1 Table and S1 Fig for a listing and the effects, respectively, of the other tested angiogenic inhibitors) [25]. Media changes with IWP2 were performed every other day. At the end of 5 days post-plating, the cells were processed and imaged as described above.

### Cytotoxicity Assay

hSVF cells were cultured in different IWP2 concentrations (0, 6.25, 12.5, 25μM, and 50μM in hSVF GM) as described above and assayed for cytotoxicity using CCK-8 (Dojindo Molecular Technologies) according to the manufacturer’s instructions. Absorbance was measured using a Synergy 4 plate reader (BioTek).

### Gene and Protein Expression

Samples from P1 hSVF cell cultures were acquired on days 1, 3, and 5 and processed for mRNA using QIAShredder and RNeasy kits (Qiagen) according to the manufacturer’s instructions. cDNA production, RT-PCR, and gel electrophoresis were all conducted as previously described [24]. RT-PCR reactions (primers can be found in S2 Table) utilized the following settings: 25 cycles, with denaturation at 95°C, annealing at 59°C, and extension at 70°C for 30 seconds. Detected bands were normalized to *GAPDH* via densitometry analysis with Image J software (NIH). For detection of protein expression, wells were prepared for fluorescence microscopy as previously described [24]. Cells were permeabilized in 0.5% Triton X-100 (MP Biomedicals) for 10 minutes, blocked in 5% goat serum (Thermo Fisher) for 1 hour, and incubated with anti-human Wnt5a antibody (Abcam) at 1:250 and 4°C overnight. Goat anti-Rabbit 594 secondary antibody (Thermo Fisher) was added the following day at 1:1000 for 1 hour at RT (refer to S3 Table for further details). Wnt5a was visualized using an IX81 inverted microscope (Olympus) and MPE FV1000 confocal microscope (Olympus).

### Exogenous Wnt5a Treatment

hSVF was plated and allowed to adhere overnight in hSVF GM. The next day, hSVF cells were treated with 25μM IWP2 and varying concentrations of recombinant human Wnt5a (0, 0.75, 7.5, 75, and 150 ng/ml; R&D Systems) for an additional 4 days. As a comparison, recombinant human Wnt3a was added to 25μM IWP2 in hSVF GM at the same concentrations. Media changes were performed every other day. At the end of day 5 post-plating, all of the wells were labeled with UEA1-Fl and quantified as previously described.

### Wnt5a-Specific Neutralization and In Vivo Analysis

Based on the in vitro results, a representative donor hSVF population was used for Wnt5a neutralization and in vivo experimentation. P1 hSVF cells were cultured as described in hSVF GM containing 0, 5, 10, or 20μg/ml of anti-Wnt5a antibody or normal goat IgG isotype control antibody (R&D Systems; of note, though the anti-Wnt5a antibody used here is specific for mouse and rat, others have successfully used it to neutralize human Wnt5a [26, 27]). Media changes occurred every other day. At the end of day 3, all of the wells were labeled with UEA1-Fl and quantified as previously described.

To validate Wnt5a’s role in vivo, hSVF was incorporated into 3 mg/ml collagen-I constructs as previously described [22, 23]. One of three treatments was incorporated into each construct: (A) 20 μg/ml IgG isotype control antibody, (B) 20 μg/ml anti-Wnt5a antibody, or (C) 7.5 ng/ml recombinant human Wnt5a. Constructs were placed bilaterally in the subcutaneous dorsum of 9 Rag1^-/-^ immune compromised C57BL6 mice (Jackson Laboratories) as previously described [22, 23]. After 2 weeks, animals were sacrificed and the constructs explanted. Constructs were fixed in 4% PFA for 1 hour, washed, permeabilized in 0.5% Triton X-100 (MP Biomedicals) for 15 minutes, and blocked with 5% goat serum (Thermo Fisher) for 1 hour. Constructs were incubated in 1:500 UEA1-Fl overnight. The following day, they were washed and incubated with DAPI (1:10000) for 10 minutes. Imaging was conducted via fluorescence confocal microscopy (Olympus MPE FV1000).

### 2-D (In Vitro) Image Analysis

Fluorescence images were analyzed using Image J software (NIH) with the Neuron J plugin (ImageScience.org). Images were assessed for total EC network length and EC area. Sheets of EC were not considered to be discrete segments and were omitted from total EC network length calculations, though they were incorporated into EC network area measurements. With regards to calculating the EC area, images were first given a color threshold to identify all UEA1^+^ structures. A size threshold of 0.065 cm^2^ was then applied to remove background noise. Resulting areas in a field-of-view were automatically quantified; images containing blatant outliers (for e.g., UEA1^+^ particles not associated with a vascular structure) were manually excluded from EC area calculations. Regarding calculations and graphical representation, we normalized in vitro rhWnt5a, rhWnt3a, and anti-Wnt5a quantitative data to their respective 0 ng/ml or 0 μg/ml treatment controls. This was done to account for the inherent differences between three different hSVF isolations from three patients. Examples of length and area measurements are shown in S2 Fig.

### 3D (In Vivo) Image Analysis

Z-stacks of confocal images were combined into a 3D composite image using AMIRA 6.0 software (FEI Visualization Sciences Group). After measuring UEA1^+^ volume, we converted UEA1^+^ signals into a skeleton and measured total EC network length, segments, and nodes. Network segments were considered to be structures with easily discernible origins and end points. The longest measurable vascular structure devoid of branching vessels was considered to be one segment. Likewise, each branch point was considered to be one node. All image manipulation and measurement was conducted according to instructions present in the AMIRA 6.0 User Guide.

### Statistics

Passaging, angiogenic inhibitor, rhWnt5a and rhWnt3a experiments were conducted in triplicate, with one experiment per donor lipoaspirate. Anti-Wnt5a and in vivo experimentation utilized cells from one representative donor. Error is presented as the mean ± standard error of the mean (SEM). Significance was determined using the Student’s t-test (p < 0.05) and verified by one-way ANOVA with follow-up Holm-Sidak or Tukey comparison tests, as determined by SigmaPlot (Systat Software). Outliers were identified using Chauvinet’s criterion [28].

## Results

### hSVF EC Networks Are Reduced with Increasing Culture

**Fig 1:**
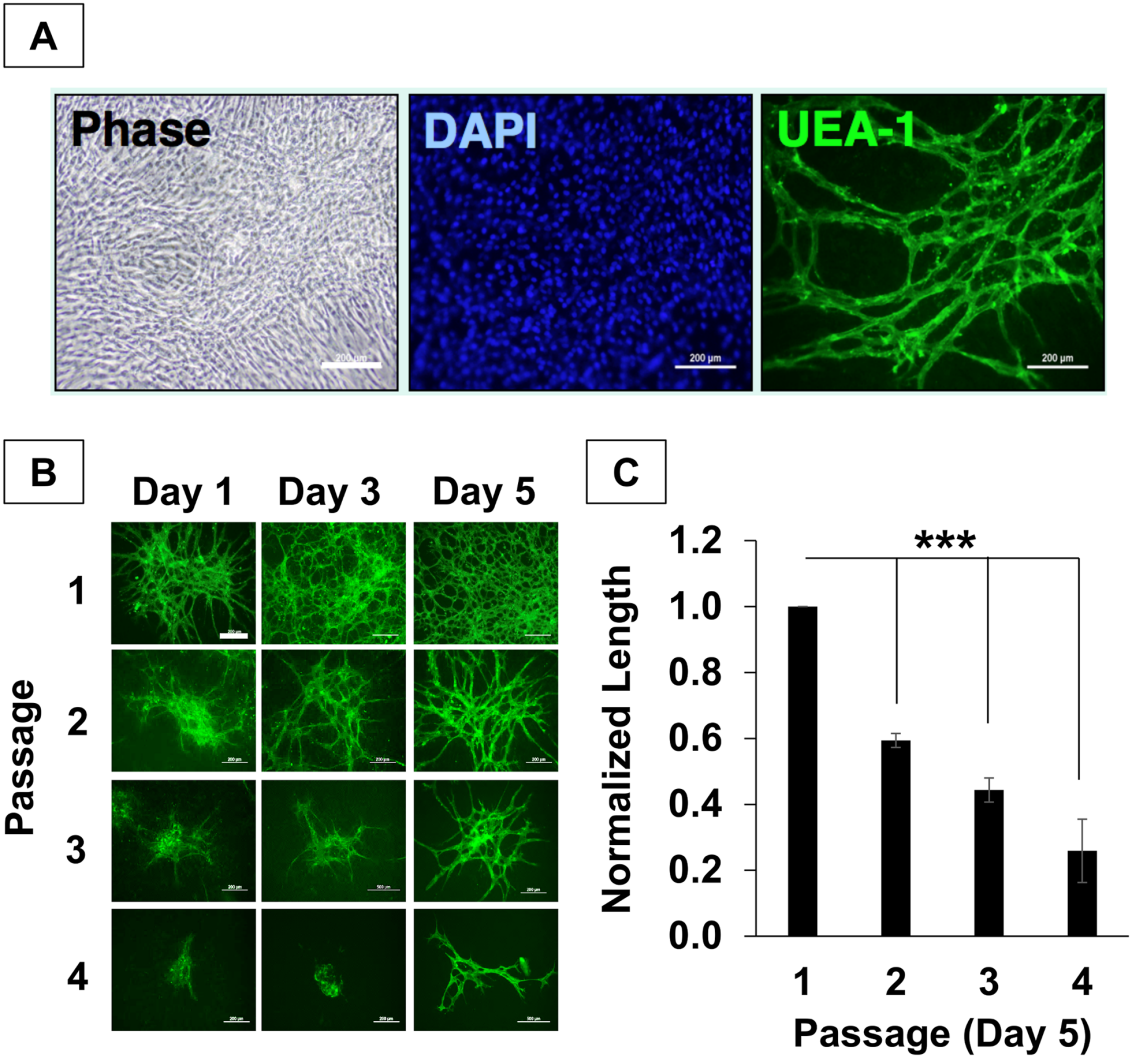
Culture reduces hSVF vascularization potential. **(A)** Within 5 days of culture, hSVF displayed complex UEA1^+^ EC networks. *Scale = 10x*, *200μm*. **(B)** hSVF networks increase in complexity over time in low passage culture, but rapidly lose EC networking capacity with subsequent passaging. *Scale = 10x*, *200μm*. n = 3 donors. **(C)** The average total hSVF EC length at day 5 was quantified and normalized to measurements of P1, day 5. Significant decreases in total hSVF EC length are seen by day 5 as a function of increasing passage (***p ≤ 0.001). Bars shown as mean ± S.E.M.

hSVF was cultured to assess potential mechanisms regulating its vascular self-assembly in vivo [22, 29]. After several days of culture, we observed the appearance of network-like structures in our cultures. Labeling of the cells with UEA1 (a lectin that binds human endothelium) and DAPI confirmed the presence of extensive EC networks in hSVF surrounded by an abundant stromal support system (Fig 1A).

Based on prior experience working with rodent and human SVF cells, we observed that high-passage SVF cells lose their ability to form vasculatures in vivo [22]. Therefore, we wanted to determine if this phenomenon also occurred in vitro. We performed an analysis of EC network formation as a function of passage, using hSVF cells from passages 1 through 4. Fig 1B shows that EC network density increased within a given passage over 5 days. Conversely, the overall EC density decreased with increasing passage. We observed significant reductions in normalized EC network length at day 5 as the passage number increased (Fig 1C). Normalized to P1, which exhibited the highest total EC length, P2, P3, and P4 cells exhibited lengths that were 0.59 ± 0.02 (*p* < 0.001), 0.44 ± 0.03 (*p* < 0.001), and 0.26 ± 0.09 (*p* = 0.001) times as large, respectively. Thus, successive culturing decreases the hSVF EC networking capacity.

While lower passages of hSVF achieved a greater quantified total EC network length by day 5 (Fig 1C), this self-assembly appears to be dependent on the number of UEA1^+^ EC within the culture at time of plating. As our analysis depends on having quantifiable EC networks, all subsequent in vitro and in vivo experiments utilized P1 cells due to their observed higher EC networking potential.

### Wnt Signaling Is Implicated in hSVF EC Network Self-Assembly

**Fig 2:**
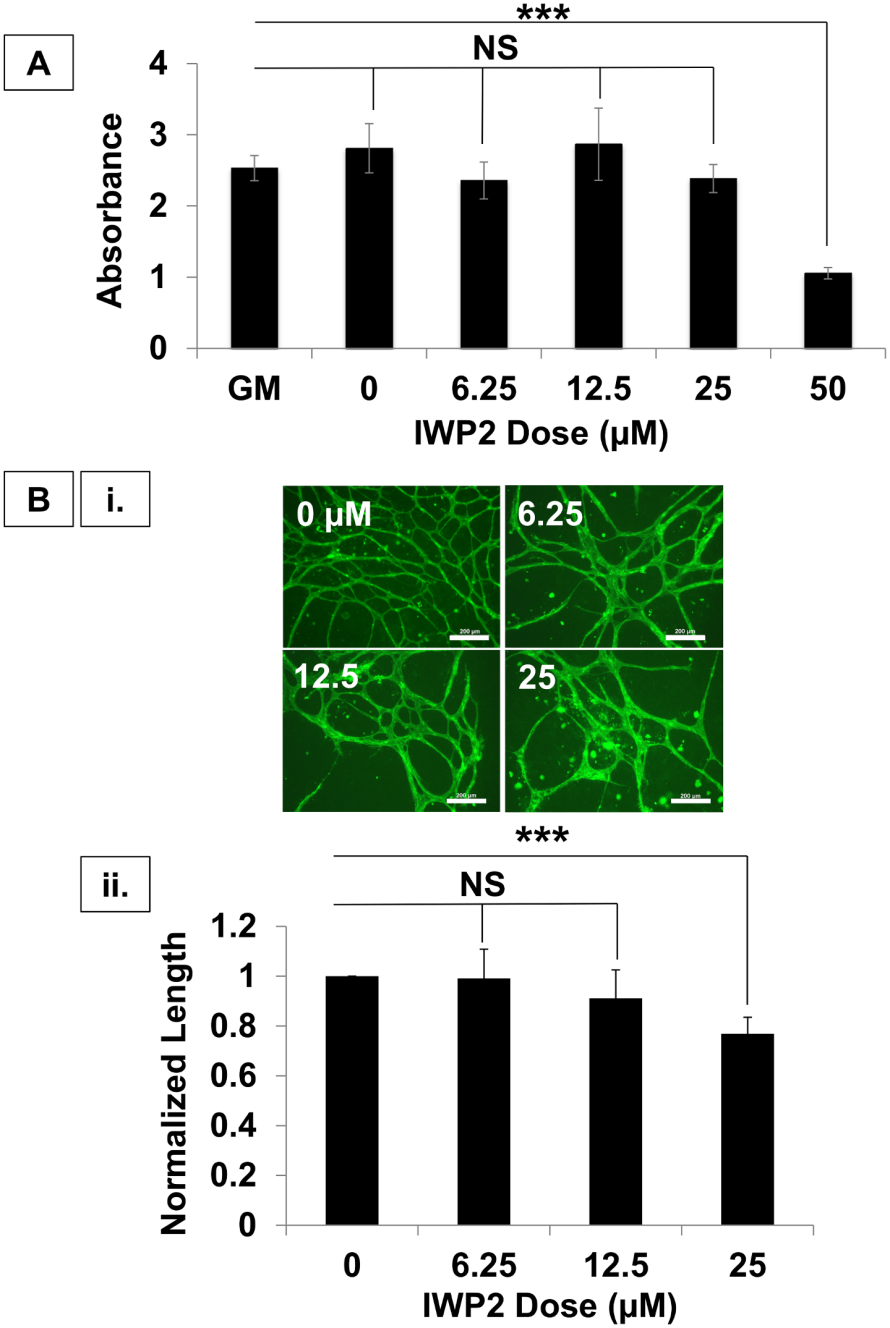
Wnt mediates hSVF vascular self-assembly. **(A)** The CCK-8 cell cytotoxicity assay demonstrated that IWP2 concentrations ≤ 25μM were not cytotoxic, whereas 50μM was cytotoxic (***p ≤ 0.001). All subsequent experiments utilized 25μM. **(B) (i.)** Increasing IWP2 concentrations reduced hSVF UEA1^+^ EC networking. *Scale = 10x*, *200μm*. **(ii.)** 25μM IWP2 significantly decreased the total network length with respect to control (***p ≤ 0.001). n = 3 donors. NS = not significant. Bars shown as mean ± S.E.M.

To identify the mechanisms regulating the self-assembly of adipose-derived hSVF into EC networks, we treated hSVF cultures with angiogenic inhibitors for the following signaling pathways: VEGF-R2 (ZM323881), PDGF-Rβ (AG1296), HGF receptor / cMet (SU11274), the TGF-β type-I receptor/Alk5 (SB431542) (S1 Fig and S1 Table), and Wnt palmitoylation (IWP2) (Fig 2). In our preliminary tests, IWP2 significantly decreased the total EC length at the two highest concentrations (25 and 50μM; data not shown). Because the role of Wnt signaling in vessel assembly is not as well characterized as it is in cancer, stem cell, and developmental biology, we focused our efforts here.

Before proceeding, we sought to determine if our preliminary findings were due to IWP2-induced cytotoxicity (Fig 2A). At 50μM of IWP2, cell death was determined to be due to cytotoxic effects (1.05 ± 0.08 AU with *p* ≤ 0.001), while the 25μM and lower concentrations yielded absorbance readings that were not significantly different from control cultures. The 25μM IWP2 dose was used for all subsequent experiments.

We next wanted to determine the effect of varying IWP2 concentrations on hSVF-EC network formation (Fig 2B.i). Treatment of hSVF with 6.25 or 12.5μM IWP2 showed no changes in EC network formation compared to control cultures. However, treating hSVF cells with 25μM IWP2 significantly reduced their total EC network length, yielding a total length only 0.75 ± 0.12 times that of the untreated control (*p* < 0.001; Fig 2B.ii). These findings demonstrate that the inhibition of endogenous hSVF Wnt signaling has a downstream effect of reducing EC self-assembly and total EC network length.

### WNT5A and FZD4 Are Expressed in hSVF

**Fig 3:**
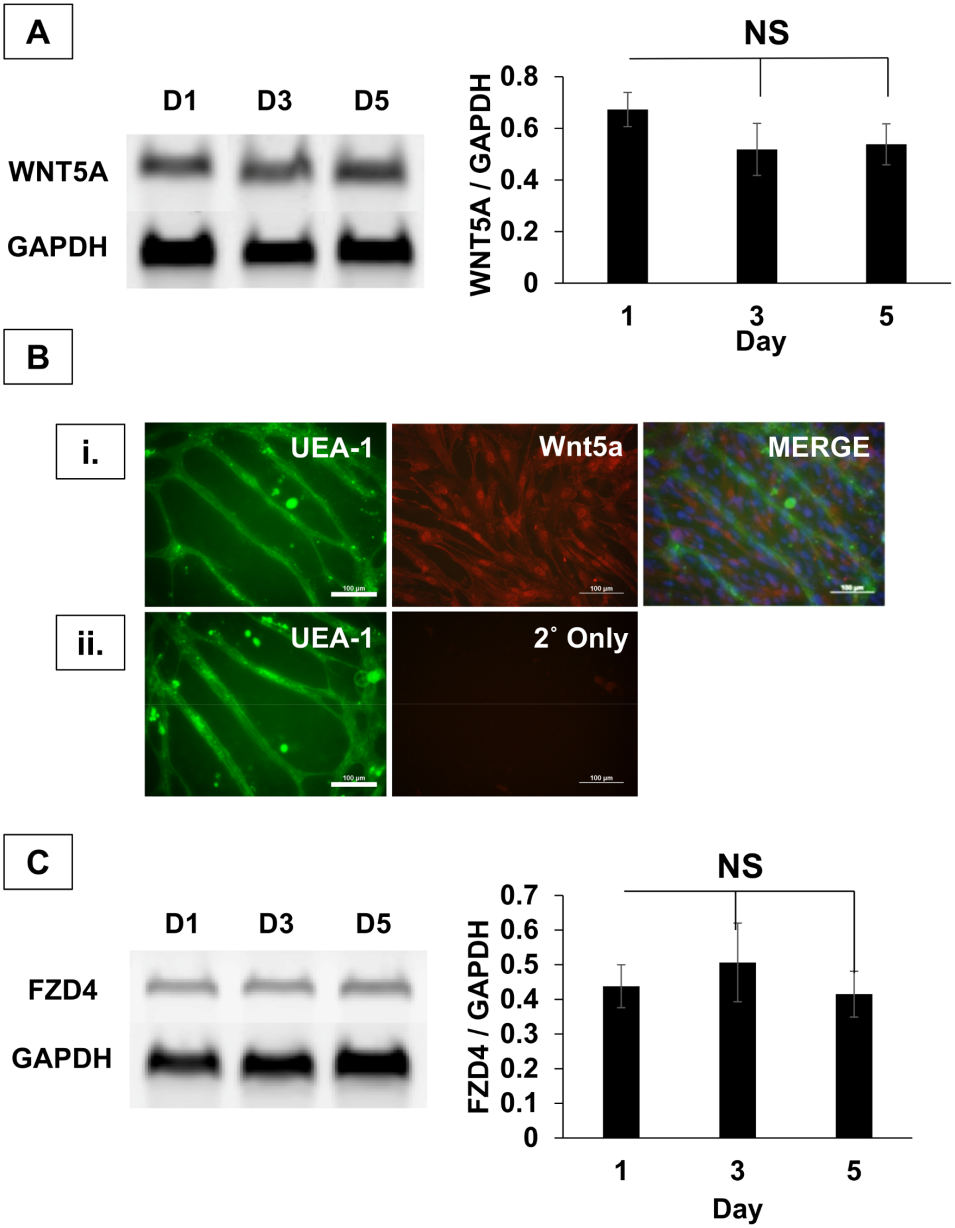
*WNT5A* and *FZD4* are highly expressed in hSVF cultures. **(A)** RT-PCR demonstrates the robust expression of *WNT5A* at days 1, 3 and 5. Normalization of *WNT5A* expression to *GAPDH* indicates uniform expression of transcripts over 5 days. n = 3 donors. **(B) (i.)** EC were labeled with UEA1 (green), nuclei with DAPI (blue), and the entire culture with anti-Wnt5a 1° + AF594 2° antibodies (red). Robust Wnt5a expression was seen throughout the culture. *Scale = 20x*, *100μm*. **(ii.)** A negative control run in parallel used only the AF594 2° antibody to rule out non-specific fluorophore binding. *Scale = 20x*, *100μm*. **(C)** *WNT5A* receptor expression was also tested by RT-PCR (also see S3B Fig). *FZD4* was consistently expressed, while densitometry shows no change in *FZD4* transcripts over 5 days. n = 3 donors. NS = not significant. Bars shown as mean ± S.E.M.

Since IWP2 blocks Wnt palmitoylation [25] and all Wnt isoforms are palmitoylated [30], we next wanted to determine which Wnt isoforms were expressed in hSVF. We used mRNA isolated from hSVF cultures at days 1, 3 and 5 to identify Wnt isoforms associated with angiogenesis via PCR [31–40] (S3A Fig). In each experimental replicate, *WNT5A* mRNA was consistently present with stable levels of expression at all time points (Fig 3A). To determine if Wnt5a protein was present in hSVF, we immunolabeled cultures with anti-Wnt5a antibody and UEA1^+^ (Fig 3B). We found that Wnt5a was abundant in the hSVF culture (Fig 3B.i), and that Wnt5a was not detectable in the fluorescent secondary antibody-only control (Fig 3B.ii).

Given that Wnt5a was present in hSVF cultures, we next wanted to determine which Wnt5a receptors were expressed in hSVF (S3B Fig). Wnt5a has been shown to interact with a number of receptors, including Frizzled-4 [41], -5 [26, 36, 42], and ROR2 [43, 44]. The hSVF mRNA isolated from hSVF cultures at days 1, 3, and 5 was also assessed for the expression of these receptors. In certain replicates, we noticed weak expression of *FZD5* and *ROR2* (S3B Fig). However, in all replicates, *FZD4* was consistently expressed at all time points (Fig 3C). These findings confirm that Wnt5a and its receptor, *FZD4*, are expressed within cultured hSVF and suggest a possible mechanism for regulating hSVF EC vascular self-assembly.

### Recombinant Wnt5a Mediates the Self-Assembly of hSVF EC Networks in IWP2-Treated Cultures

**Fig 4:**
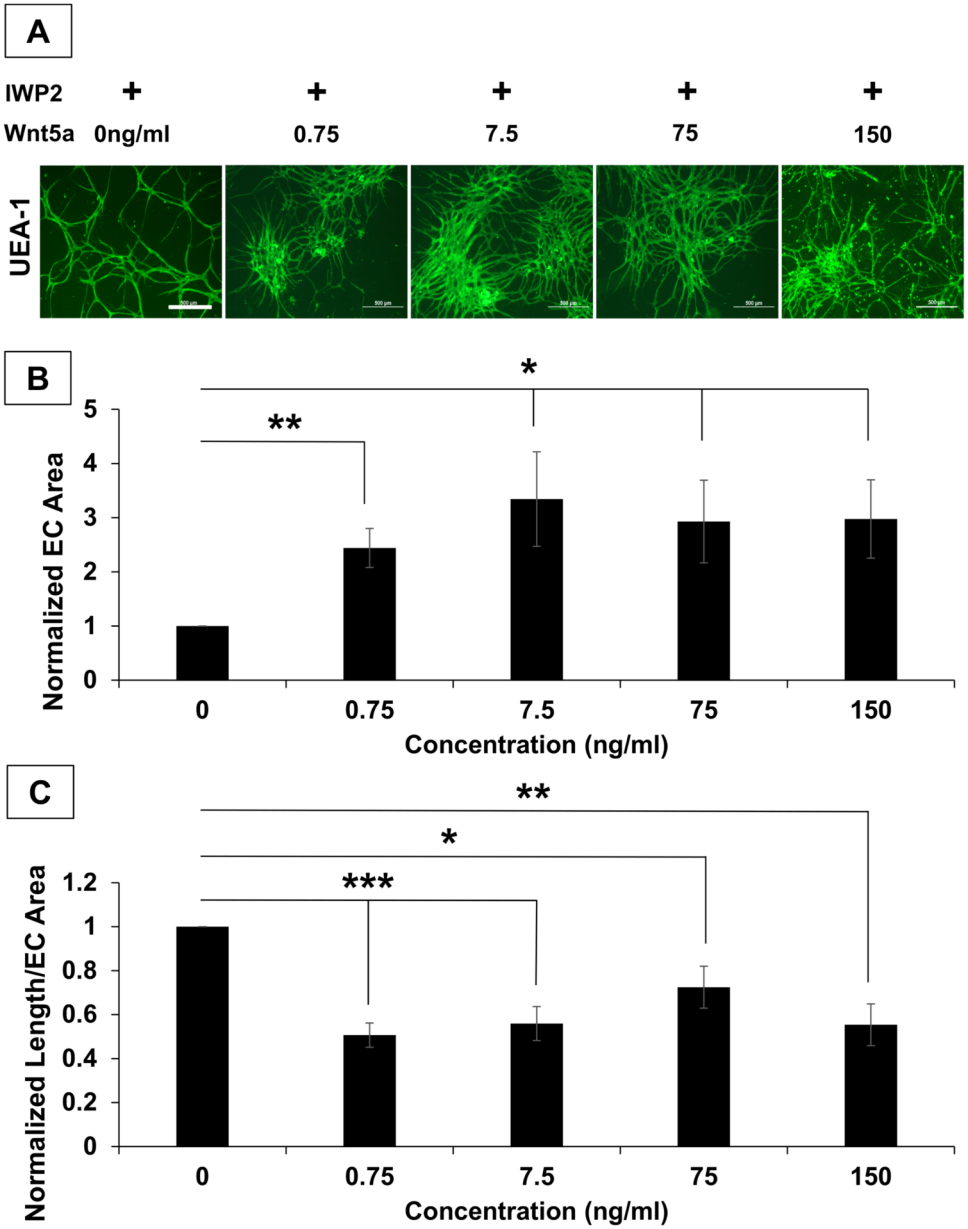
Exogenous recombinant Wnt5a mediates hSVF vascular self-assembly during IWP2 inhibition. hSVF cultures were treated with 25μM IWP2 and given increasing concentrations of rhWnt5a. **(A)** Representative images of UEA1^+^ (green) hSVF EC networks are seen for all conditions. *Scale Bar = 4x*, *500μm*. **(B)** rhWnt5a significantly increased the total UEA1^+^ area compared to the control (*p ≤ 0.05; **p ≤ 0.01). **(C)** Compared to the 0 ng/ml rhWnt5a control, supplemental rhWnt5a produced areas of hSVF EC sheeting, which is characterized by lower ratios of total EC length to EC area. n = 3 donors. Bars shown as mean ± S.E.M.

To determine the specific role of Wnt5a in the self-assembly of hSVF EC networks, we exposed hSVF cultures to a combination of 25μM IWP2 and varying concentrations (0–150 ng/ml) of exogenously supplied recombinant human Wnt5a protein (rhWnt5a; Fig 4). Since IWP2 (a) inhibits the release of all endogenous Wnt isoforms from hSVF cells without affecting cognate receptors and (b) impairs hSVF EC networking, we wanted to determine if the selective addition of rhWnt5a alone would restore hSVF EC network assembly.

After five days of exposure to rhWnt5a and IWP2, hSVF cultures were labeled with UEA1 (Fig 4A), allowing us to quantify the magnitude of hSVF EC networking. As expected, the addition of 25μM IWP2 alone (negative control, with no supplemental rhWnt5a) inhibited EC network assembly. Supplementing the cultures with as little as 0.75 ng/ml rhWnt5a yielded significant increases in UEA1^+^ EC area (Fig 4B) compared to cultures given IWP2 alone, resulting in a 2.44 ± 0.36-fold increase (*p* ≤ 0.01). The 7.5 ng/ml concentration elicited the largest increase in area, measuring 3.34 ± 0.87 times (*p* ≤ 0.05) larger than cultures treated with IWP2 alone, while 75 ng/ml elicited a 2.93 ± 0.76-fold increase (*p* ≤ 0.05) and 150 ng/ml yielded an increase of 2.98 ± 0.72 fold (*p* ≤ 0.05). ANOVA analysis further indicated that treatment with any concentration of rhWnt5a was significant compared to controls lacking rhWnt5a, but that there were no significant differences between the various rhWnt5a treatments.

Interestingly, while addition of rhWnt5a significantly increased UEA1^+^ EC area in the presence of IWP2, it also promoted the aggregation of EC into sheet-like structures. To quantitatively characterize this, the total EC network length was measured in each field-of-view and normalized this length to the measured EC area in that same field-of-view (Fig 4C). Compared to cultures given IWP2 alone, the addition of 0.75 ng/ml rhWnt5a yielded ratios that were only 0.51 ± 0.06 times as large (*p* ≤ 0.001). Similarly, the 7.5, 75, and 150 ng/ml concentrations exhibited ratios that were 0.56 ± 0.08 (*p* ≤ 0.001), 0.72 ± 0.1 (*p* ≤ 0.05), and 0.55 ± 0.09 (*p* ≤ 0.001) times that seen with the control. Thus, considering that rhWnt5a increased EC area and reduces the ratio of total EC length to EC area, rhWnt5a appears to specifically play a role in modulating hSVF vascular network assembly in an EC proliferation-dependent process.

**Fig 5:**
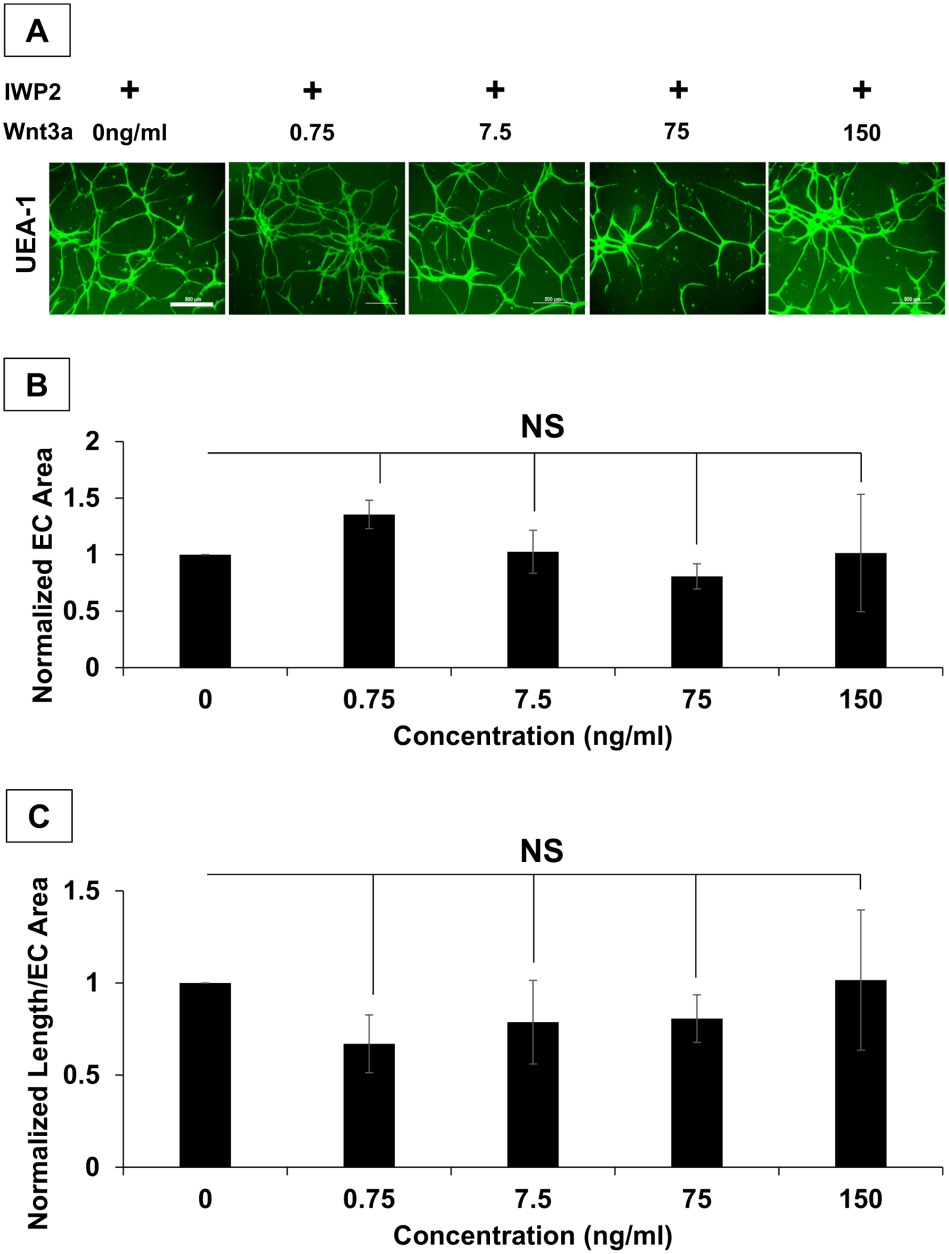
IWP2-treated hSVF EC networks are not affected by supplemental rhWnt3a. **(A)** hSVF was treated with 25μM IWP2 for 5d and given varying concentrations of rhWnt3a. Representative images of UEA1^+^ (green) hSVF EC networks are seen for all conditions. *Scale Bar = 4x*, *500μm*. **(B)** rhWnt3a supplementation yielded no significant changes to network area compared to the IWP2 control. **(C)** The normalization of total UEA1^+^ EC network length to the total UEA1^+^ EC area was not significantly affected by exogenous rhWnt3a addition. n = 3 donors. NS = not significant. Bars shown as mean ± S.E.M.

Wnt signaling is divided into canonical and non-canonical pathways, which utilizes β-catenin or non-β-catenin pathways, respectively, to transduce the signal [45]. Wnt5a can bind to a variety of receptors but is generally categorized as a non-canonical Wnt [27, 46]. To determine if the same effect on hSVF EC networking could be produced by a different Wnt isoform, we repeated the experiment above using the prototypical canonical Wnt, Wnt3a, at the same concentrations as those used for the rhWnt5a in vitro experiment (Fig 5A). Here we determined that Wnt3a supplementation did not significantly increase networking area (Fig 5B) or the ratio of EC network length to EC area of IWP2-treated cultures (Fig 5C). Further, ANOVA analysis confirmed that treatment with any concentration of Wnt3a did not yield a significant outcome. These findings suggest that Wnt5a alone can partially restore the assembly of hSVF EC vascular structures when endogenous Wnt release is prevented. It also demonstrates that the effect on network assembly is not necessarily mediated by all Wnt isoforms, since Wnt3a substitution was ineffective.

### Wnt5a Mediates hSVF Microvascular Self-Assembly In Vivo

Our data supports that Wnt5a plays a specific role in hSVF microvascular self-assembly in vitro. Therefore, we wanted to determine its role in the self-assembly that we and others have reported in vivo [21, 22, 24]. We initially exposed hSVF cultures to varying concentrations (0–20 μg/ml) of Wnt5a neutralizing antibody (referred to as anti-Wnt5a) [26] in vitro to determine an appropriate concentration for use in vivo (S4 Fig). Compared to the negative control (0 μg/ml), there were no significant differences were observed with 5 or 10 μg/ml anti-Wnt5a. However, treatment with 20 μg/ml anti-Wnt5a significantly reduced the total EC length (0.68 ± 0.06 fold, *p* ≤ 0.05; S4A Fig) and segment number (0.52 ± 0.09 fold, *p* ≤ 0.05; S4B Fig). The addition of an IgG control antibody at the same concentrations did not affect total EC length or segment number (data not shown).

**Fig 6:**
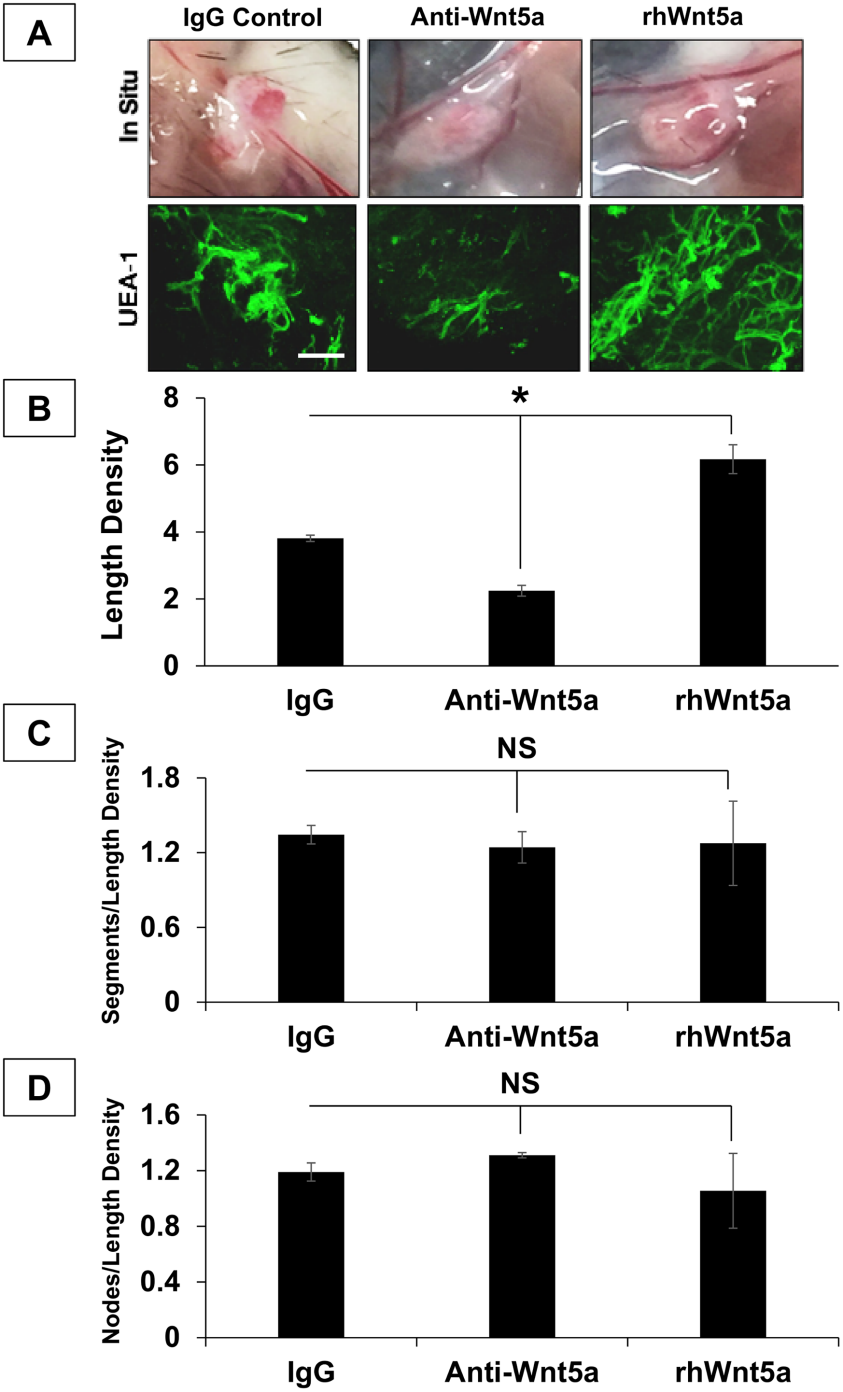
Wnt5a drives hSVF EC microvascular assembly in vivo. **(A)** 3D collagen-I constructs containing hSVF an IgG isotype control, Wnt5a neutralizing antibody (anti-Wnt5a), or rhWnt5a (3 animals per treatment; 1 representative donor cell line was used) were implanted for 2w. Images show 2w constructs in situ. Constructs were then labeled with UEA1 (green) and imaged by confocal microscopy. *Scale bar = 20x*, *100μm*. **(B)** Anti-Wnt5a treatment significantly reduced the length density compared to the IgG control (*p ≤ 0.05), while rhWnt5a addition significantly increased this ratio (*p ≤ 0.05). **(C)** Normalization of segment number to length density showed no significant affect with either the anti-Wnt5a or the rhWnt5a treatments compared to IgG controls. **(D)** The ratio of node number to length density also showed no significant difference between the IgG control, the anti-Wnt5a treatment, and treatment with rhWnt5a. NS = not significant. Bars shown as mean ± S.E.M.

Based on these in vitro findings, we utilized 20 μg/ml anti-Wnt5a in subsequent in vivo experiments to determine if Wnt5a modulated hSVF microvascular assembly in that setting as well. hSVF cells were incorporated into 3D collagen-I constructs containing either 20 μg/ml IgG isotype control antibody, 20 μg/ml anti-Wnt5a neutralizing antibody, or 7.5 ng/ml rhWnt5a (Fig 6). After two weeks of implantation, the constructs were explanted, processed and analyzed as described in the Materials and Methods (Fig 6A). Each experimental condition was quantified for length density (i.e., a ratio of the total EC length to the 3D scan volume; Fig 6B) as well as the ratios of segment (Fig 6C) and node (Fig 6D) numbers to length density. Thus, length density is a measure of assembly, while the segment and node ratios serve to characterize hSVF vascular complexity.

IgG controls exhibited an average length density of (3.81 ± 0.1) x 10^−5^ μm/μm^3^. Treatment with anti-Wnt5a significantly reduced the length density ((2.25 ± 0.16) x 10^−5^ μm/μm^3^; *p* ≤ 0.05), while rhWnt5a significantly increased this ratio ((6.17 ± 0.43) x 10^−5^ μm/μm^3^; *p* ≤ 0.05). Thus, compared to the IgG control, anti-Wnt5a reduced total vascular length by approximately 41 ± 5.6%, while rhWnt5a increased overall vascular length by approximately 62 ± 15%.

With regards to vascular complexity, the addition of anti-Wnt5a to 3D implants did not significantly affect the ratio of segments to length density ((1.24 ± 0.13) x 10^7^ segments/μm/μm^3^; *p* = 0.56) compared to IgG controls ((1.34 ± 0.07) x 10^7^ segments/μm/μm^3^). Anti-Wnt5a also bore no significant effect on the ratio of nodes to length density, with (1.31 ± 0.02) x 10^7^ nodes/μm/μm^3^ (*p* = 0.22) compared to the (1.24 ± 0.07) x 10^7^ nodes/μm/μm^3^ seen with the IgG control. Additionally, rhWnt5a did not significantly influence complexity either ((1.28 ± 0.34) x 10^7^ segments/μm/μm^3^ (*p* = 0.86) and (1.06 ± 0.27) x 10^7^ nodes/μm/μm^3^ (*p* = 0.67)). These results also correlate with the qualitative observation that 3D implants did not contain EC sheet-like aggregations.

ANOVA analysis indicated that the anti-Wnt5a and rhWnt5a treatments only imparted significant effects on the EC length within a 3D construct. Thus, this data demonstrates that in a 3D implant, the supplementation of rhWnt5a in vivo significantly increased the overall length of the hSVF microvasculature, while the specific blockade of Wnt5a with a neutralizing antibody significantly reduced hSVF cells’ capability to form a microcirculation. Together, these findings support a role for Wnt5a in mediating hSVF microvascular self-assembly in vivo.

## Discussion

The main and novel findings of this report are that (a) the complete heterogeneous cellular mixture that comprises human adipose SVF (hSVF) spontaneously forms endothelial networks in vitro, but loses this capacity with serial cell culture due to a reduction in system endothelium, (b) in vivo hSVF microvascular self-assembly can be modeled in vitro by the endogenous heterogeneous hSVF cellular mixture and (c) the self-assembly of hSVF microvascular networks in vivo is regulated by Wnt5a signaling that mediates proliferation but not microvascular complexity.

Koh and colleagues demonstrated that the formation of vascular networks by mouse SVF depended on the presence of EC [21]. We previously reported that the removal of EC from rat SVF yields a significantly altered vascular interaction [22]. Here, we show that the depletion of endogenous UEA1^+^ hSVF-EC reduces its spontaneous EC networking. While it seems obvious that the depletion of endogenous EC from SVF results in a decreased capacity for spontaneous vascular self-assembly, this finding emphasizes that the heterogeneous mixture of the different cell types within SVF work together as an interdependent system. It also suggests that once endogenous EC are depleted, their function is not significantly replaced by other differentiating cells residing within the same system. These findings support the hypothesis that the heterogeneity of SVF is crucial to its biological activity. However, the mechanisms regulating this functionality are poorly defined.

Koh and colleagues demonstrated VEGF was required for SVF vascular assembly [21]. CD31^-^/CD34^+^ cells (isolated from SVF by differential attachment on untreated tissue culture plastic) were shown to produce multiple angiogenic growth factors, including VEGF, and provide perivascular support when cultured or implanted with exogenous microvascular EC [16]. Our data supports these findings and also demonstrates that Wnt5a regulates hSVF microvascular self-assembly in vivo. Though Wnt biology has been extensively studied in other contexts, its functions in EC biology and angiogenesis have only recently been investigated [40, 47–50], with defined roles for canonical and non-canonical Wnt isoforms [36, 40, 47] in vascularization. Our data demonstrates that Wnt5a is highly expressed in hSVF. Though the cell population(s) that produces Wnt5a remains to be identified, it most likely originates from non-endothelial cells and acts in a paracrine manner. We also showed robust expression of *FZD4* receptor transcripts in hSVF. Wnt5a has been shown to utilize this receptor in EC [41], but it can also signal through FZD5 [36] and ROR2 [44, 51]. Thus, the downstream signaling events also need to be defined. Lastly, though more work is required to understand how the hSVF cellular system functions to self-assemble a microvasculature, our data identifies Wnt5a signaling as a target to potentiate this process.

Much work as been performed to isolate and characterize the adipose stromal cell (ASC) [15, 52, 53]–partly because of its similarity in function to bone marrow mesenchymal stem cells (MSC) and also because of its relative ease of access [54–58]. ASC are isolated by serial culture and have been shown to be adipogenic, chondrogenic, osteogenic and myogenic, similar to bone marrow MSC [15]. We have demonstrated that by using the entire endogenous SVF cell system, we can generate a microcirculation that supports the engraftment and function of implanted parenchymal cells [10, 24, 59]. Though certain lineages contained within adipose SVF may be advantageous for specific applications, our data illustrates the utility of the entire endogenous SVF cell mixture as a potential therapeutic system [60].

In this report, our goal was to define mechanisms controlling hSVF spontaneous EC microvascular network formation in vivo. Our findings are novel, as this is the first identification of Wnt5a as a regulator of hSVF vascular assembly. This work underscores the fact that adipose SVF is a potential regenerative cell system. Understanding how the mixture of heterogeneous cell types within hSVF works together could enhance efforts to better vascularize tissues or promote the engraftment of engineered therapeutic cells [22, 24].

## Supporting Information

S1 Fig hSVF EC network quantification after inhibitor treatment. hSVF was treated with small molecule inhibitors to identify potential pathways mediating vascular self-assembly. For each inhibitor tested, UEA1^+^ network length was normalized to the untreated control (also see S1 Table). The VEGF-R2 and PDGF-Rβ inhibitors significantly reduced total hSVF EC network length at their two highest concentrations (*p ≤ 0.05), whereas no significant differences were seen with any concentration of the TGF-β/Alk5 or HGF/cMet inhibitors.
(TIFF)

S2 Fig EC network tracing and area determination. **(A)** Image J was used to convert standard UEA1^+^ EC images to 8-bit. Images were then traced to measure total EC length using the NeuronJ plugin. **(B)** 8-bit images were also used for area measurement. Images were processed for size threshold (shown in red) and enumerated as described in the Materials and Methods.
(TIFF)

S3 Fig *WNT* and *WNT5A*-Receptor PCR Screening. **(A)** Day 1 hSVF cDNA was initially screened by PCR using primers for Wnt isoforms associated with angiogenesis. Of these, *WNT5A* was most strongly expressed, while *WNT7B* was expressed to a lesser degree (arrows). **(B)** Once *WNT5A* was identified, *WNT5A* receptors underwent similar screening, with a focus on *FZD4*, *FZD5*, and *ROR2*. *FZD4* was consistently expressed for all experiments, while *FZD5* and *ROR2* expression was inconsistent.
(TIFF)

S4 Fig Wnt5a neutralizing antibody inhibits hSVF total EC length and segments. The largest concentration of anti-Wnt5a (20 μg/ml) significantly reduced the **(A)** hSVF EC total length (*p ≤ 0.05) and **(B)** number of segments (*p ≤ 0.05). This concentration was used to examine Wnt5a’s role in hSVF vascular self-assembly *in vivo*. In both (A) and (B), values obtained for length and segments with each treatment were normalized to the 0 μg/ml control. NS = not significant.
(TIFF)

S1 Table List of Inhibitors Used. (DOCX)

S2 Table List of Primers Used in RT-PCR. (DOCX)

S3 Table List of Antibodies/Lectins/Stains Used. (DOCX)

## References

[1] Langer R. Tissue engineering: perspectives, challenges, and future directions. Tissue Eng. 2007;13(1):1–2. 10.1089/ten.2006.0219 . 17518575

[2] Naderi H, Matin MM, Bahrami AR. Review paper: critical issues in tissue engineering: biomaterials, cell sources, angiogenesis, and drug delivery systems. J Biomater Appl. 2011;26(4):383–417. 10.1177/0885328211408946 . 21926148

[3] Ilan N, Mahooti S, Madri JA. Distinct signal transduction pathways are utilized during the tube formation and survival phases of in vitro angiogenesis. J Cell Sci. 1998;111 (Pt 24):3621–31. . 9819353 10.1242/jcs.111.24.3621

[4] Boyd NL, Nunes SS, Jokinen JD, Krishnan L, Chen Y, Smith KH, et al Microvascular mural cell functionality of human embryonic stem cell-derived mesenchymal cells. Tissue Eng Part A. 2011;17(11–12):1537–48. 10.1089/ten.TEA.2010.0397 21284534 PMC3098949

[5] Boyd NL, Nunes SS, Krishnan L, Jokinen JD, Ramakrishnan VM, Bugg AR, et al Dissecting the role of human embryonic stem cell-derived mesenchymal cells in human umbilical vein endothelial cell network stabilization in three-dimensional environments. Tissue Eng Part A. 2013;19(1–2):211–23. 10.1089/ten.tea.2011.0408 22971005 PMC3530951

[6] Davis GE, Black SM, Bayless KJ. Capillary morphogenesis during human endothelial cell invasion of three-dimensional collagen matrices. In Vitro Cell Dev Biol Anim. 2000;36(8):513–9. Epub 2001/01/10. 10.1290/1071-2690(2000)036<0513:CMDHEC>2.0.CO;2 . 11149750

[7] Stratman AN, Schwindt AE, Malotte KM, Davis GE. Endothelial-derived PDGF-BB and HB-EGF coordinately regulate pericyte recruitment during vasculogenic tube assembly and stabilization. Blood. 2010;116(22):4720–30. Epub 2010/08/27. blood-2010-05-286872 [pii]10.1182/blood-2010-05-286872. 10.1182/blood-2010-05-286872 20739660 PMC2996127

[8] Levenberg S, Rouwkema J, Macdonald M, Garfein ES, Kohane DS, Darland DC, et al Engineering vascularized skeletal muscle tissue. Nat Biotechnol. 2005;23(7):879–84. 15965465 10.1038/nbt1109

[9] Gong Z, Niklason LE. Small-diameter human vessel wall engineered from bone marrow-derived mesenchymal stem cells (hMSCs). FASEB J. 2008. 10.1096/fj.07-087924 PMC2605790 18199698

[10] Nunes SS, Maijub JG, Krishnan L, Ramakrishnan VM, Clayton LR, Williams SK, et al Generation of a functional liver-tissue mimic using adipose stromal vascular fraction cell-derived vasculatures. Sci Rep. 2013;3(2141):1–7. 10.1038/srep02141 PubMed Central PMCID: PMC3701895. PMC3701895 23828203

[11] Crandall DL, Hausman GJ, Kral JG. A review of the microcirculation of adipose tissue: anatomic, metabolic, and angiogenic perspectives. Microcirculation. 1997;4(2):211–32. Epub 1997/06/01. . 9219215 10.3109/10739689709146786

[12] Rupnick MA, Panigrahy D, Zhang CY, Dallabrida SM, Lowell BB, Langer R, et al Adipose tissue mass can be regulated through the vasculature. Proc Natl Acad Sci U S A. 2002;99(16):10730–5. Epub 2002/08/01. 10.1073/pnas.162349799 12149466 PMC125027

[13] Rodbell M. Metabolism of Isolated Fat Cells. I. Effects of Hormones on Glucose Metabolism and Lipolysis. J Biol Chem. 1964;239:375–80. Epub 1964/02/01. . 14169133

[14] Williams SK, Jarrell BE, Rose DG, Pontell J, Kapelan BA, Park PK, et al Human microvessel endothelial cell isolation and vascular graft sodding in the operating room. Ann Vasc Surg. 1989;3(2):146–52. Epub 1989/04/01. . 2765356 10.1016/S0890-5096(06)62008-6

[15] Zuk PA, Zhu M, Mizuno H, Huang J, Futrell W, Katz AJ, et al Multilineage cells from human adipose tissue: implications for cell-based therapies. Tissue Eng Part A. 2001;7(2):211–28. 10.1089/107632701300062859 11304456

[16] Traktuev DO, Merfeld-Clauss S, Li J, Kolonin M, Arap W, Pasqualini R, et al A population of multipotent CD34-positive adipose stromal cells share pericyte and mesenchymal surface markers, reside in a periendothelial location, and stabilize endothelial networks. Circ Res. 2008;102(1):77–85. 10.1161/CIRCRESAHA.107.159475 . 17967785

[17] Williams SK, Wang TF, Castrillo R, Jarrell BE. Liposuction-derived human fat used for vascular graft sodding contains endothelial cells and not mesothelial cells as the major cell type. J Vasc Surg. 1994;19(5):916–23. Epub 1994/05/01. S0741521494003071 [pii]. . 8170048 10.1016/s0741-5214(94)70019-2

[18] Gimble JM, Bunnell BA, Chiu ES, Guilak F. Concise review: Adipose-derived stromal vascular fraction cells and stem cells: let's not get lost in translation. Stem Cells. 2011;29(5):749–54. 10.1002/stem.629 . 21433220

[19] Han J, Koh YJ, Moon HR, Ryoo HG, Cho CH, Kim I, et al Adipose tissue is an extramedullary reservoir of functional hematopoetic stem and progenitor cells. Blood. 2010;115(5):957–64. 19897586 10.1182/blood-2009-05-219923

[20] Yoshimura K, Shigeura T, Matsumoto D, Sato T, Takaki Y, Aiba-Kojima E, et al Characterization of freshly isolated and cultured cells derived from the fatty and fluid portions of liposuction aspirates. J Cell Physiol. 2006;208(1):64–76. 10.1002/jcp.20636 . 16557516

[21] Koh YJ, Koh BI, Kim H, Joo HJ, Jin HK, Jeon J, et al Stromal vascular fraction from adipose tissue forms profound vascular network through the dynamic reassembly of blood endothelial cells. Arterioscler Thromb Vasc Biol. 2011;31(5):1141–50. 10.1161/ATVBAHA.110.218206 . 21393582

[22] Nunes SS, Maijub JG, Krishnan L, Ramakrishnan VM, Clayton LR, Williams SK, et al Generation of a functional liver tissue mimic using adipose stromal vascular fraction cell-derived vasculatures. Sci Rep. 2013;3:2141 10.1038/srep02141 ; PubMed Central PMCID: 3701895. 23828203 PMC3701895

[23] Maijub JG, Boyd NL, Dale JR, Hoying JB, Morris ME, Williams SK. Concentration Dependent Vascularization of Adipose Stromal Vascular Fraction Cells. Cell Transplant. 2014 10.3727/096368914X685401 . 25397993

[24] Ramakrishnan VM, Yang JY, Tien KT, McKinley TR, Bocard BR, Maijub JG, et al Restoration of physiologically responsive low-density lipoprotein receptor-mediated endocytosis in genetically deficient induced pluripotent stem cells. Sci Rep. 2015;5:13231 10.1038/srep13231 26307169 PMC4549683

[25] Chen B, Dodge ME, Tang W, Lu J, Ma Z, Fan CW, et al Small molecule-mediated disruption of Wnt-dependent signaling in tissue regeneration and cancer. Nat Chem Biol. 2009;5(2):100–7. 10.1038/nchembio.137 19125156 PMC2628455

[26] Blumenthal A, Ehlers S, Lauber J, Buer J, Lange C, Goldmann T, et al The Wingless homolog WNT5A and its receptor Frizzled-5 regulate inflammatory responses of human mononuclear cells induced by microbial stimulation. Blood. 2006;108(3):965–73. 10.1182/blood-2005-12-5046 . 16601243

[27] Cheng CW, Yeh JC, Fan TP, Smith SK, Charnock-Jones DS. Wnt5a-mediated non-canonical Wnt signalling regulates human endothelial cell proliferation and migration. Biochem Biophys Res Commun. 2008;365(2):285–90. 10.1016/j.bbrc.2007.10.166 . 17986384

[28] Lin L, Sherman PD. Cleansing data the Chauvinet way. SESUG Proc. 2007:1–11.

[29] Montesano R, Orci L, Vassalli P. In vitro rapid organization of endothelial cells into capillary-like networks is promoted by collagen matrices. J Cell Biol. 1983;97:1648–52. 6630296 10.1083/jcb.97.5.1648 PMC2112683

[30] Willert K, Brown JD, Danenberg E, Duncan AW, Weissman IL, Reya T, et al Wnt proteins are lipid-modified and can act as stem cell growth factors. Nature. 2003;423:448–52. 12717451 10.1038/nature01611

[31] Reis M, Czupalla CJ, Ziegler N, Devraj K, Zinke J, Seidel S, et al Endothelial Wnt/beta-catenin signaling inhibits glioma angiogenesis and normalizes tumor blood vessels by inducing PDGF-B expression. J Exp Med. 2012;209(9):1611–27. 10.1084/jem.20111580 22908324 PMC3428944

[32] Gherghe CM, Duan J, Gong J, Rojas M, Klauber-Demore N, Majesky M, et al Wnt1 is a proangiogenic molecule, enhances human endothelial progenitor function, and increases blood flow to ischemic limbs in a HGF-dependent manner. FASEB J. 2011;25(6):1836–43. 10.1096/fj.10-172981 21321190 PMC3219217

[33] Klein D, Demory A, Peyre F, Kroll J, Geraud C, Ohnesorge N, et al Wnt2 acts as an angiogenic growth factor for non-sinusoidal endothelial cells and inhibits expression of stanniocalcin-1. Angiogenesis. 2009;12(3):251–65. 10.1007/s10456-009-9145-5 . 19444628

[34] Daneman R, Agalliu D, Zhou L, Kuhnert F, Kuo CJ, Barres BA. Wnt/ß-catenin signaling is required for CNS, but not non-CNS, angiogenesis. Proc Natl Acad Sci U S A. 2009;106(2):641–6. 10.1073/pnas.0901563106 19129494 PMC2626756

[35] Jover B, Girardot D, de Courtois Roy de Vacquieres F, Casellas D, Moles JP. Wnt-4 potently inhibits capillary outgrowth from rat aorta in 3D culture. Fundam Clin Pharmacol. 2013;27(5):465–70. 10.1111/j.1472-8206.2012.01044.x . 22607657

[36] Arderiu G, Espinosa S, Pena E, Aledo R, Badimon L. Monocyte-secreted Wnt5a interacts with FZD5 in microvascular endothelial cells and induces angiogenesis through tissue factor signaling. J Mol Cell Biol. 2014;6(5):380–93. 10.1093/jmcb/mju036 . 25240054

[37] Murdoch CE, Bachschmid MM, Matsui R. Regulation of neovascularization by S-glutathionylation via the Wnt5a/sFlt-1 pathway. Biochem Soc Trans. 2014;42(6):1665–70. 10.1042/BST20140213 . 25399587 PMC4934611

[38] Melgar-Lesmes P, Edelman ER. Monocyte-endothelial cell interactions in the regulation of vascular sprouting and liver regeneration in mouse. J Hepatol. 2015 10.1016/j.jhep.2015.05.011 . 26022689 PMC4575901

[39] Ekström EJ, Bergenfelz C, von Bülow V, Serifier F, Carlemalm E, Jönsson G, et al WNT5A induces release of exosomes containing pro-angiogenic and immunosuppressive factors from malignant melanoma cells. Molecular Cancer. 2014;13(88):1–15. 24766647 10.1186/1476-4598-13-88 PMC4022450

[40] Korn C, Scholz B, Hu J, Srivastava K, Wojtarowicz J, Arnsperger T, et al Endothelial cell-derived non-canonical Wnt ligands control vascular pruning in angiogenesis. Development. 2014;141(8):1757–66. 10.1242/dev.104422 . 24715464

[41] Bian WJ, Miao WY, He SJ, Wan ZF, Luo ZG, Yu X. A novel Wnt5a-Frizzled4 signaling pathway mediates activity-independent dendrite morphogenesis via the distal PDZ motif of Frizzled4. Dev Neurobiol. 2015;75(8):805–22. 10.1002/dneu.22250 . 25424568

[42] Sen M, Chamorro M, Reifert J, Corr M, Carson DA. Blockade of Wnt-5A/frizzled 5 signaling inhibits rheumatoid synoviocyte activation. Arthritis Rheum. 2001;44(4):772–81. 11315916 10.1002/1529-0131(200104)44:4<772::AID-ANR133>3.0.CO;2-L

[43] Oishi I, Suzuki H, Onishi N, Takada R, Kani S, Ohkawara B, et al The receptor tyrosine kinase Ror2 is involved in non-canonical Wnt5a/JNK signalling pathway. Genes Cells. 2003;8:645–54. 12839624 10.1046/j.1365-2443.2003.00662.x

[44] Liu Y, Rubin B, Bodine PV, Billiard J. Wnt5a induces homodimerization and activation of Ror2 receptor tyrosine kinase. J Cell Biochem. 2008;105(2):497–502. 10.1002/jcb.21848 . 18615587

[45] Angers S, Moon RT. Proximal events in Wnt signal transduction. Nat Rev Mol Cell Biol. 2009;10(7):468–77. 10.1038/nrm2717 . 19536106

[46] Catalan V, Gomez-Ambrosi J, Rodriguez A, Perez-Hernandez AI, Gurbindo J, Ramirez B, et al Activation of noncanonical Wnt signaling through WNT5A in visceral adipose tissue of obese subjects is related to inflammation. J Clin Endocrinol Metab. 2014;99(8):E1407–17. 10.1210/jc.2014-1191 . 24840810

[47] Dejana E. The role of wnt signaling in physiological and pathological angiogenesis. Circ Res. 2010;107(8):943–52. 10.1161/CIRCRESAHA.110.223750 . 20947863

[48] Maschauchán TNH, Agalliu D, Vorontchikhina M, Ahn A, Parmalee N, Li C, et al Wnt5a signaling induces proliferation and survival of endothelial cells in vitro and expression of MMP-1 and Tie-2. Mol Biol Cell. 2006;17:5163–72. 17035633 10.1091/mbc.E06-04-0320 PMC1679681

[49] Goodwin AM, Sullivan KM, D'Amore PA. Cultured endothelial cells display endogenous activation of the canonical Wnt signaling pathway and express multiple ligands, receptors, and secreted modulators of Wnt signaling. Dev Dyn. 2006;235(11):3110–20. 10.1002/dvdy.20939 . 17013885

[50] Newman AC, Hughes CCW. Macrophages and angiogenesis: a role for Wnt signaling. Vasc Cell. 2012;4(13):1–7. 22938389 10.1186/2045-824X-4-13 PMC3479425

[51] Himmelreich N, Kaufmann LT, Steinbeisser H, Korner C, Thiel C. Lack of phosphomannomutase 2 affects Xenopus laevis morphogenesis and the non-canonical Wnt5a/Ror2 signalling. J Inherit Metab Dis. 2015;38(6):1137–46. 10.1007/s10545-015-9874-0 . 26141167

[52] Zuk PA, Zhu M, Ashjian P, De Ugarte DA, Huang JI, Mizuno H, et al Human adipose tissue is a source of multipotent stem cells. Mol Biol Cell. 2002;13(12):4279–95. Epub 2002/12/12. 10.1091/mbc.E02-02-0105 12475952 PMC138633

[53] Lin CS, Xin ZC, Deng CH, Ning H, Lin G, Lue TF. Defining adipose tissue-derived stem cells in tissue and in culture. Histol Histopathol. 2010;25(6):807–15. Epub 2010/04/09. . 20376787 10.14670/HH-25.807

[54] De Ugarte DA, Morizono K, Elbarbary A, Alfonso Z, Zuk PA, Zhu M, et al Comparison of multi-lineage cells from human adipose tissue and bone marrow. Cells Tissues Organs. 2003;174(3):101–9. Epub 2003/07/02. 10.1159/000071150 CTO2003174003101 [pii]. . 12835573

[55] Izadpanah R, Trygg C, Patel B, Kriedt C, Dufour J, Gimble JM, et al Biologic properties of mesenchymal stem cells derived from bone marrow and adipose tissue. J Cell Biochem. 2006;99(5):1285–97. Epub 2006/06/24. 10.1002/jcb.20904 . 16795045 PMC4048742

[56] Kern S, Eichler H, Stoeve J, Kluter H, Bieback K. Comparative analysis of mesenchymal stem cells from bone marrow, umbilical cord blood, or adipose tissue. Stem Cells. 2006;24(5):1294–301. Epub 2006/01/18. 2005–0342 [pii] 10.1634/stemcells.2005-0342 . 16410387

[57] Noel D, Caton D, Roche S, Bony C, Lehmann S, Casteilla L, et al Cell specific differences between human adipose-derived and mesenchymal-stromal cells despite similar differentiation potentials. Exp Cell Res. 2008;314(7):1575–84. Epub 2008/03/08. S0014-4827(07)00589-7 [pii] 10.1016/j.yexcr.2007.12.022 . 18325494

[58] Zhu Y, Liu T, Song K, Fan X, Ma X, Cui Z. Adipose-derived stem cell: a better stem cell than BMSC. Cell Biochem Funct. 2008;26(6):664–75. Epub 2008/07/19. 10.1002/cbf.1488 . 18636461

[59] Maijub JG, Boyd NL, Dale JR, Hoying JB, Morris ME, Williams SK. Concentration-dependent vascularization of adipose stromal vascular fraction cells. Cell Transplant. 2015;24(10):2029–39. 10.3727/096368914X685401 25397993

[60] Morris ME, Beare JE, Reed RM, Dale JR, LeBlanc AJ, Kaufman CL, et al Systemically delivered adipose stromal vascular fraction cells disseminate to peripheral artery walls and reduce vasomotor tone through a CD11b+ cell-dependent mechanism. Stem Cells Transl Med. 2015;4(4):369–80. 10.5966/sctm.2014-0252 25722428 PMC4367510

