# Supplementary figures and images for: Wnt5a Regulates the Assembly of Human Adipose Derived Stromal Vascular Fraction-Derived Microvasculatures
Source: PLoS One. 2016 Mar 10;11(3):e0151402. doi: 10.1371/journal.pone.0151402 (PMC4786226; doi:10.1371/journal.pone.0151402)

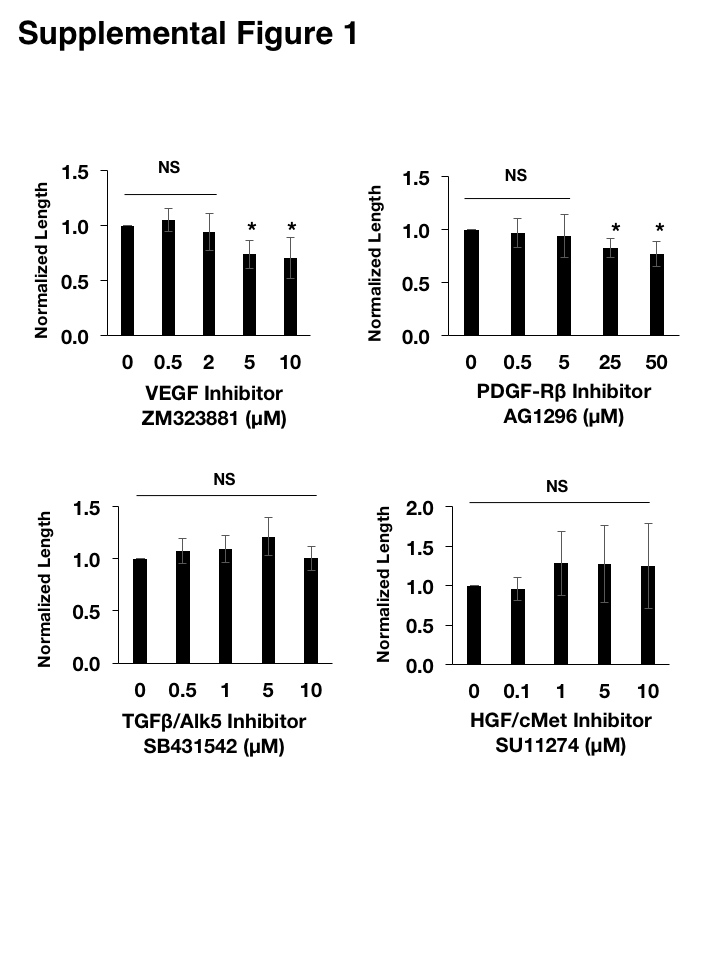

Supplement: S1 Fig — hSVF was treated with small molecule inhibitors to identify potential pathways mediating vascular self-assembly. For each inhibitor tested, UEA1+ network length was normalized to the untreated control (also see S1 Table). The VEGF-R2 and PDGF-Rβ inhibitors significantly reduced total hSVF EC network length at their two highest concentrations (*p ≤ 0.05), whereas no significant differences were seen with any concentration of the TGF-β/Alk5 or HGF/cMet inhibitors. (TIFF) [file pone.0151402.s001.tiff]

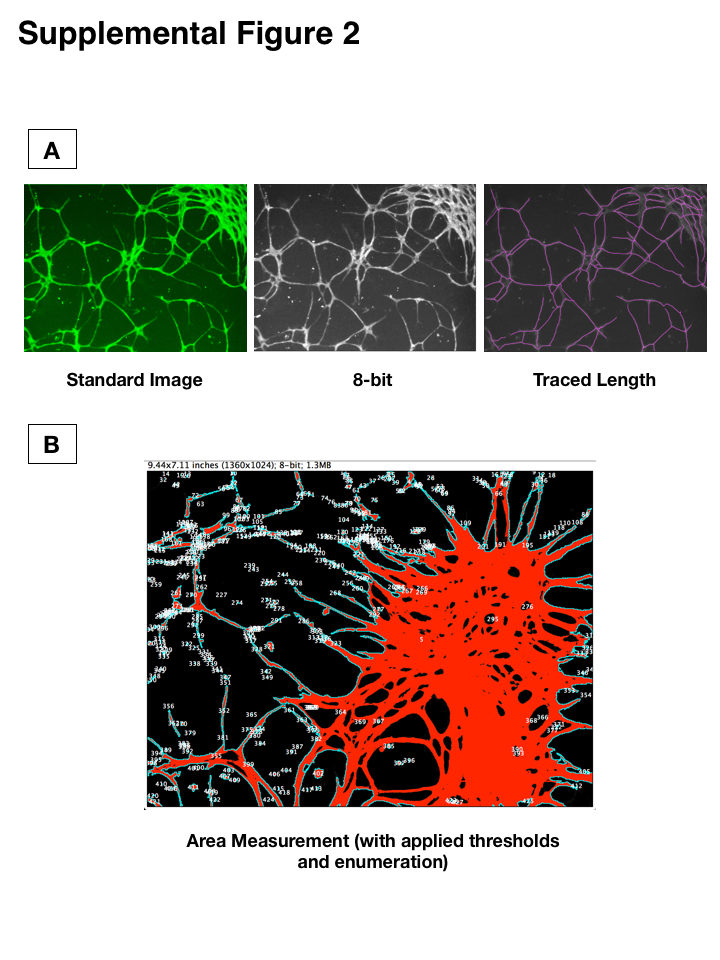

Supplement: S2 Fig — (A) Image J was used to convert standard UEA1+ EC images to 8-bit. Images were then traced to measure total EC length using the NeuronJ plugin. (B) 8-bit images were also used for area measurement. Images were processed for size threshold (shown in red) and enumerated as described in the Materials and Methods. (TIFF) [file pone.0151402.s002.tiff]

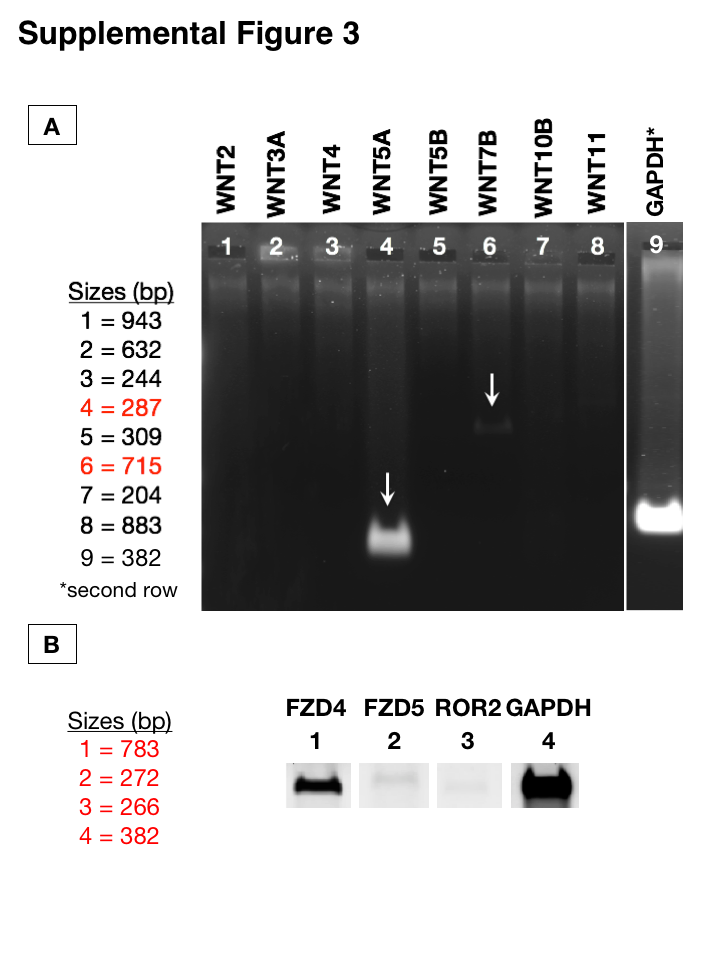

Supplement: S3 Fig — (A) Day 1 hSVF cDNA was initially screened by PCR using primers for Wnt isoforms associated with angiogenesis. Of these, WNT5A was most strongly expressed, while WNT7B was expressed to a lesser degree (arrows). (B) Once WNT5A was identified, WNT5A receptors underwent similar screening, with a focus on FZD4, FZD5, and ROR2. FZD4 was consistently expressed for all experiments, while FZD5 and ROR2 expression was inconsistent. (TIFF) [file pone.0151402.s003.tiff]

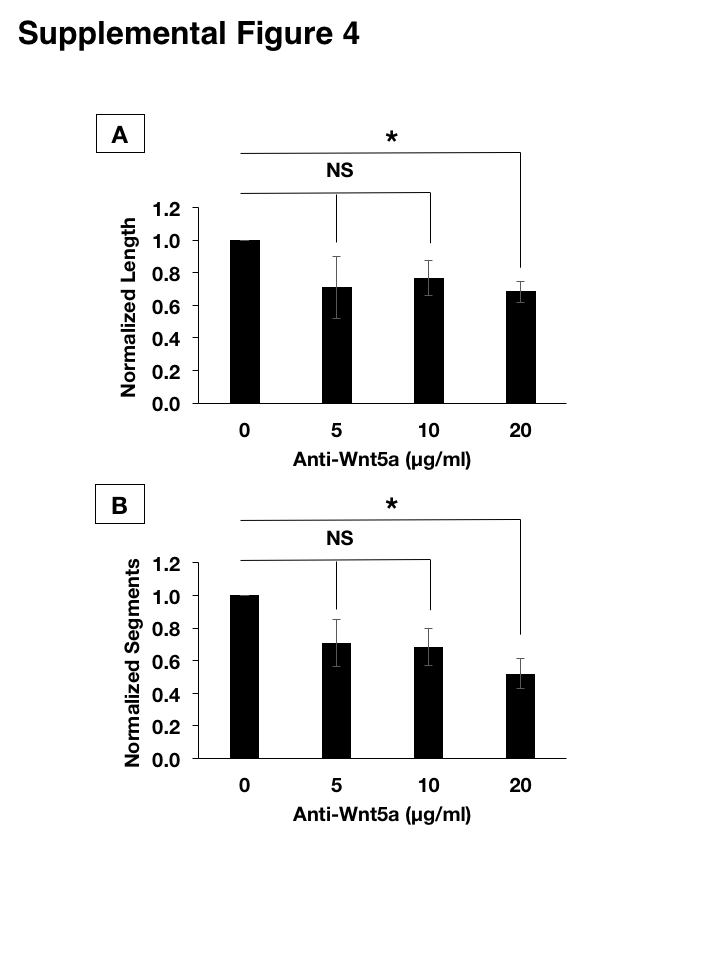

Supplement: S4 Fig — The largest concentration of anti-Wnt5a (20 μg/ml) significantly reduced the (A) hSVF EC total length (*p ≤ 0.05) and (B) number of segments (*p ≤ 0.05). This concentration was used to examine Wnt5a’s role in hSVF vascular self-assembly in vivo. In both (A) and (B), values obtained for length and segments with each treatment were normalized to the 0 μg/ml control. NS = not significant. (TIFF) [file pone.0151402.s004.tiff]
