# Supplementary material for: Wnt5a Regulates the Assembly of Human Adipose Derived Stromal Vascular Fraction-Derived Microvasculatures
Source: PLoS One. 2016 Mar 10;11(3):e0151402. doi: 10.1371/journal.pone.0151402 (PMC4786226; doi:10.1371/journal.pone.0151402)
Supplement: S1 Table — (DOCX) [file pone.0151402.s005.docx]

**Supplemental Table 1. List of Inhibitors Used**

| **Inhibitor** | **Designation** | **Vendor (Catalog No).** | **Dose Range** | **Significant Effect Observed?** |
| --- | --- | --- | --- | --- |
| **Wnt Palmitoylation** | IWP2 | Tocris (3533) | 0, 6.25, 12.5, 25, and 50 µM | **Yes** |
| **VEGF-R2** | ZM323881 | Tocris (2475) | 0, 0.5, 2.5, 5, and 10 µM | **Yes** |
| **PDGF-Rβ** | AG1296 | Calbiochem (658551) | 0, 0.5, 5, 25, and 50 µM | **Yes** |
| **TGF-β1 / Alk-5** | SB431542 | Stemcell Technologies (72232) | 0, 0.5, 1, 5, and 10 µM | No |
| **HGF / cMet** | SU11274 | Selleck Chemicals (S1080) | 0, 0.5, 1, 5, and 10 µM | No |
