# Supplementary material for: Wnt5a Regulates the Assembly of Human Adipose Derived Stromal Vascular Fraction-Derived Microvasculatures
Source: PLoS One. 2016 Mar 10;11(3):e0151402. doi: 10.1371/journal.pone.0151402 (PMC4786226; doi:10.1371/journal.pone.0151402)
Supplement: S2 Table — (DOCX) [file pone.0151402.s006.docx]

**Supplemental Table 2. List of Primers Used in RT-PCR**

| **Gene Name** | **NCBI Reference Sequence** | **5’ 🡪 3’ Primer Sequence**  **(Forward / Reverse)** | **Product Size (bp)** | **Annealing Temp. ˚C (Forward / Reverse)** | **Expressed in hSVF?** |
| --- | --- | --- | --- | --- | --- |
| **WNT2** | NM_003391.2 | CTGGCCTTTATCGCTCGCTG / TTCATGACCACCTGGATGG | 943 | 61.49 / 60.03 | No |
| **WNT3A** | NM_033131.3 | CCTCCCTGGAGCTAGTGTCT /  GGAACCTTACAGGGGGTTGG | 632 | 60.03 / 59.96 | No |
| **WNT4** | NM_030761.4 | TCGTCTTCGCCGTCTTCTCAG /  GAGTCGAGTGTGGAGCAGTT | 244 | 62.12 / 59.68 | No |
| **WNT5A** | NM_003392.4 | AAGCAGACGTTTCGGCTACA /  TTTCCAACGTCCATCAGCGA | 287 | 59.97 / 59.97 | **Yes** |
| **WNT5B** | NM_030775.2 | CTCAAGAGAGCGAGAAGACTGG /  CCCTCCCCTATGTAGGCCAT | 309 | 60.16 / 60.18 | No |
| **WNT7B** | NM_058238.2 | GGAGGCTTCCACCTTTCTCC /  TCTCCGGTACCCAGTGTAGG | 715 | 60.03 / 60.03 | **Yes** |
| **WNT10B** | NM_003394.3 | TGAGCTCGGTGAGAGCAAAG /  TTAAACCGTGGGGAGACTGC | 204 | 60.04 / 59.96 | No |
| **WNT11** | NM_004626.2 | CACAAGACAGGCAGTGCAAC /  TGCCGAGTTCACTTGACGAG | 883 | 59.97 / 60.32 | No |
| **FZD4** | NM_012193.3 | CCAACTGGGCACTTTTTCGG / TCTAAACAGCAGACAGCGCA | 783 | 59.97 / 59.97 | **Yes** |
| **FZD5** | NM_003468.3 | TGCTTCATCTCCACGTCCAC / AGGATGACCCACCAGATGGA | 272 | 60.04 / 59.96 | **Yes** |
| **ROR2** | NM_004560.3 | TCCTTCTGCCACTTCGTCTT / TTGTAGCACTGGTGGTAGCG | 266 | 58.95 / 60.04 | **Yes** |
| **GAPDH** | NM_001289745.1 | AATCCCATCACCATCTTCC /  CATCACGCCACAGTTTCC | 382 | 54.14 / 56.43 | **Yes** |
