# Supplementary material for: Wnt5a Regulates the Assembly of Human Adipose Derived Stromal Vascular Fraction-Derived Microvasculatures
Source: PLoS One. 2016 Mar 10;11(3):e0151402. doi: 10.1371/journal.pone.0151402 (PMC4786226; doi:10.1371/journal.pone.0151402)
Supplement: S3 Table — (DOCX) [file pone.0151402.s007.docx]

**Supplemental Table 3. List of Antibodies / Lectins / Stains Used**

| **Antibody/Lectin/Stain (Concentration)** | **Manufacturer**  **(Catalog Number)** | **Purpose** |
| --- | --- | --- |
| Wnt5a (1:250) | Abcam (ab72583) | Immunocytochemistry |
| Wnt5a (20µg/ml) | R&D Systems (AF-645) | Neutralization |
| IgG (20µg/ml) | R&D Systems (AB-108-C) | Control |
| Fluorescein-Labeled Ulex Europaeus Agglutinin Type I (UEA1; 1:500) | Vector Laboratories (FL-1061) | Immunocytochemistry |
| DAPI (1:10000) | Thermo Fisher Scientific | Immunocytochemistry |
| **2˚ Antibody**  **(Concentration)** | **Manufacturer**  **(Catalog Number)** | **Purpose** |
| Goat anti-Rabbit DyLight^TM^ 594 (1:1000) | Thermo Fisher Scientific (35560) | Immunocytochemistry |
